# Selectivity in *C*-alkylation of dianions of protected 6-methyluridine

**DOI:** 10.3762/bjoc.7.143

**Published:** 2011-09-06

**Authors:** Ngoc Hoa Nguyen, Christophe Len, Anne-Sophie Castanet, Jacques Mortier

**Affiliations:** 1 Université du Maine and CNRS, Unité de chimie organique moléculaire et macromoléculaire (UMR 6011), Faculté des sciences, avenue Olivier Messiaen, 72085 Le Mans Cedex 9, France; 2 Université de technologie de Compiègne, Transformations intégrées de la matière renouvelable, EA 4297 UTC/ESCOM, 1 allée du réseau Jean-Marie Buckmaster, 60200 Compiègne, France

**Keywords:** C6-alkylation, cyclonucleosides, lithiations, 6-ω-alkenyluridines

## Abstract

A regioselective synthesis of 6-ω-alkenyluridines **3**, precursors of potent antiviral and antitumor cyclonucleosides **5**, is described. While ω-alkenyl halides do not alkylate 6-lithiouridine, compounds **3** were prepared in a regioselective manner by sequential treatment of 6-methyluridine **2** with LTMP or LDA (4 equiv) in THF at −30 °C followed by alkylation with ω-alkenyl bromides.

## Introduction

Conformationally restricted C–C bridged cyclonucleosides bearing a linkage between the sugar moiety and the nucleobase, exhibit a broad spectrum of antiviral and antitumor activities [1–4]. Cyclonucleosides are excellent tools for studying the role of the conformational parameters that are critical for the design of new nucleoside drug candidates [4–8]. These cyclic compounds are expected to have a beneficial biological impact especially toward enzymatic repair processes [9].

**Scheme 1:**
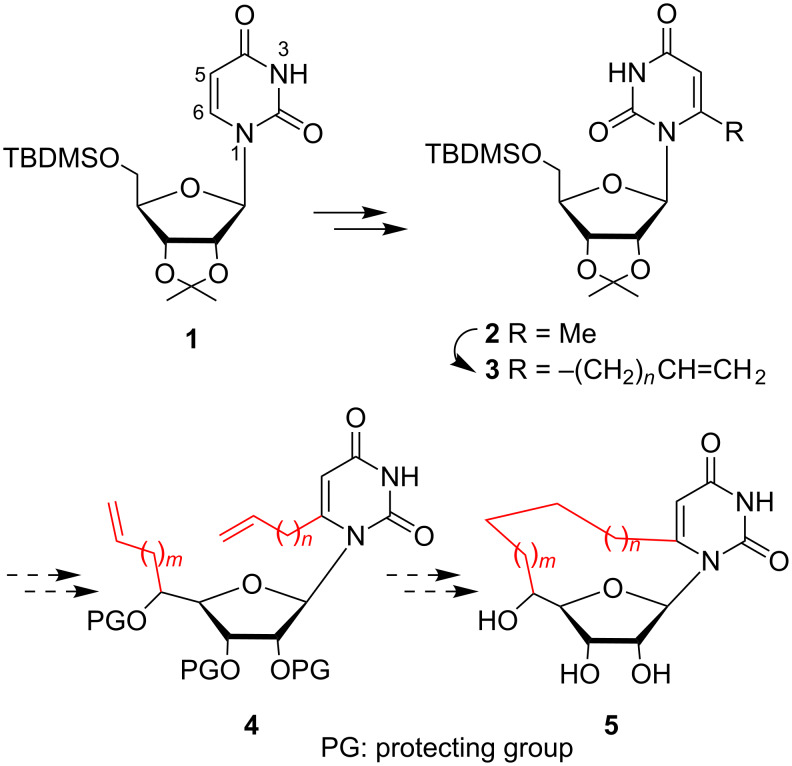
Synthesis of potent antiviral and antitumor cyclonucleosides **5**.

As part of an ongoing program directed by one of us (C. L.) toward the synthesis and development of new cyclonucleosides **5** [5–6], we envisioned that the general transformation outlined in Scheme 1 might afford a facile entry to **5** from dialkenyl precursors **4** by ring-closing metathesis [10–12]. The strategy relies on the preparation of unknown 6-ω-alkenyluridine key intermediates **3**. We report herein that sequential ring lithiation/methylation of the simple protected uridine **1** leading to **2** followed by lateral lithiation/alkylation with ω-alkenyl bromides provides a useful regioselective chain-extension procedure and an efficient route to **3**.

## Results and Discussion

**Figure 1:**
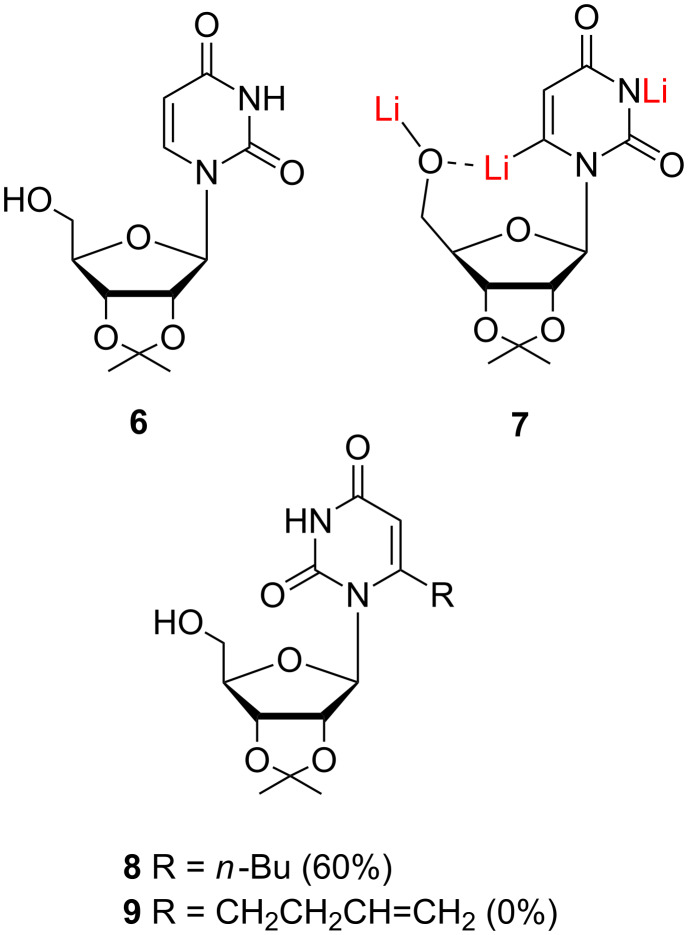
Lithiation of 2',3'-*O*-isopropylideneuridine (**6**).

Most methods for the construction of *C*-substituted nucleosides are based on ring lithiation of nucleoside derivatives followed by their reaction with appropriate electrophiles. Thus, sequential lithiation of 2',3'-*O*-isopropylideneuridine (**6**) with LDA in THF (Figure 1) and electrophilic quenching with *n*-bromobutane was reported to give 6-*n*-butyl-2',3'-*O*-isopropylideneuridine (**8**) in a regiospecific manner (60%) [13]. It seems likely that the reaction proceeds via trianion **7** where the 5'-OLi group can easily participate in the stabilization of the 6-lithio intermediate. ω-Alkenyl bromides are known to be poor electrophiles toward organolithiums [14], and indeed, **7** failed to react, in our experiments, with 4-bromo-but-1-ene to give **9**.

**Figure 2:**
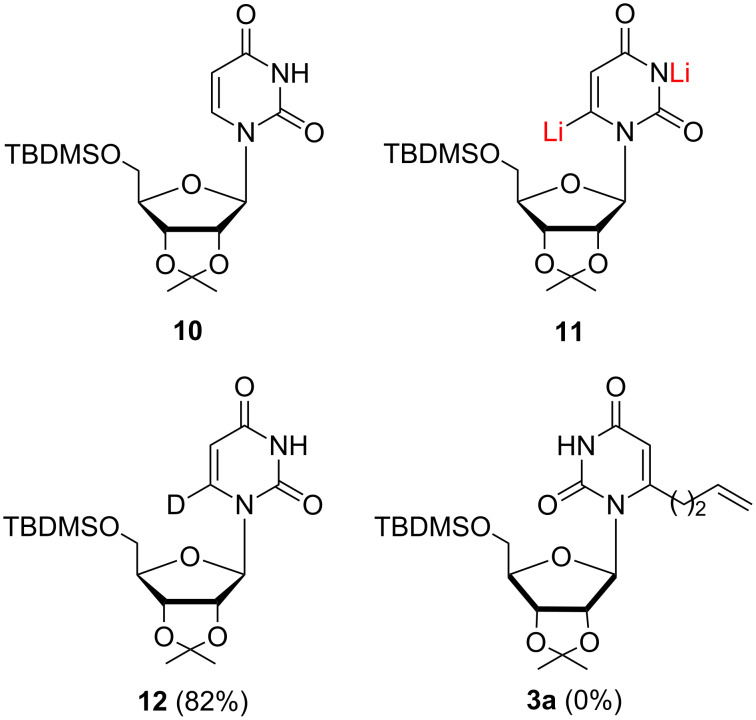
Metalation of 5'-*O*-TMDMS protected nucleoside **10**.

We then turned our attention to the metalation of the 5'-*O*-TBDMS protected nucleoside **10** (Figure 2). Treatment with LDA (5 equiv) in THF at −70 °C followed by addition of D_2_O provided **12** in 82% yield (evaluated by NMR) with exclusive deuterium incorporation at the C6 position. However, almost complete recovery of the starting material was observed when dianion **11** was allowed to react with 4-bromobut-1-ene [15]. Lithium–copper transmetallation was also attempted. Unfortunately, addition of 0.25 equiv of Li_2_CuCl_4_ [16–18] to **11** followed by quenching with 4-bromobut-1-ene failed to produce **3a**.

Consequently, lateral lithiations were examined. Lateral lithiation of benzenoid aromatics requires a stabilizing group capable of either delocalizing negative charge or stabilizing an organolithium by coordination [19–20]. Primary, allylic, and benzylic halides usually give good yields of laterally alkylated products. Secondary and acetylenic halides have been used in several instances. Successful reaction with these substrates is noteworthy since many aryllithiums arising from *ortho*-lithiation reactions do not alkylate, or give poor yields, with any halides other than iodomethane [21–24]. Competing base-induced elimination reactions are presumably observed with iodoethane and higher homologues [19–20,25–26]. It has also been proposed that poor reactivity of lithiated carbanions toward alkyl halides may result from steric hindrance [24,27]. Recently, the lateral lithiation of 4-hydroxy-6,7-dimethoxy-8-methyl-2-naphthoic acid was applied to the regioselective efficient construction of a series of 5,5'-didesisopropyl-5,5'-dialkylapogossypol derivatives that are potent pan-active inhibitors of anti-apoptotic Bcl-2 family proteins [28].

**Figure 3:**
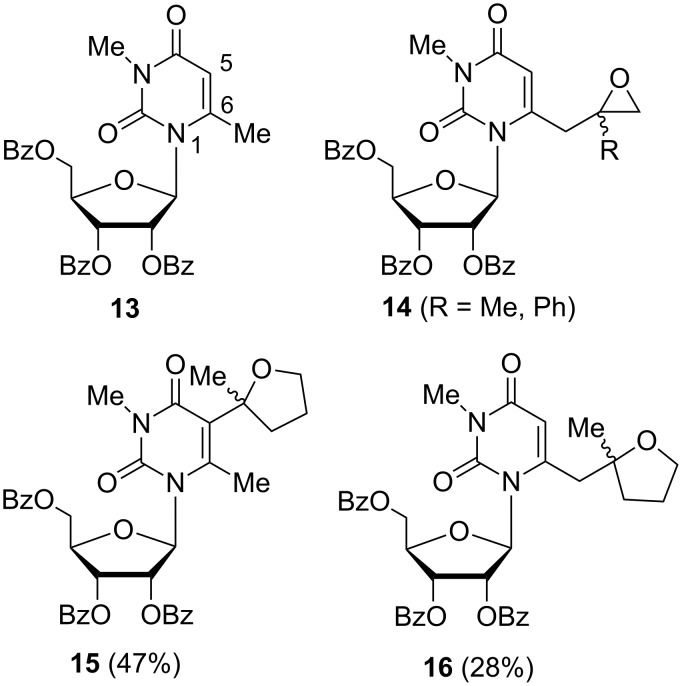
Lithiation/alkylation of 2',3',5'-tri-*O*-benzoyl-3,6-dimethyluridine (**13**) using LDA.

Literature furnishes little information regarding lateral lithiations in the nucleoside field and the data, scarce as they are, even appear to be inconsistent at first sight. Treatment of 2',3',5'-tri-*O*-benzoyl-3,6-dimethyluridine (**13**) with chloroacetone or 2-chloroacetophenone in the presence of LDA (1.2 equiv, THF, −78 °C) afforded 6-(oxiranylmethyl)uridine derivatives **14** exclusively (Figure 3) [29]. With 5-chloro-2-pentanone, the reaction led to a mixture of 5- and 6-substituted uridine regioisomers **15** and **16** in 47% and 28% yield, respectively. It was suggested that the *N*-1 sugar moiety in the *syn* orientation of the nucleoside might affect the access of a very sterically demanding electrophile, such as 5-chloro-2-pentanone, to the 6-position. This hypothesis was confirmed by a probe experiment where an even more sterically hindered racemic 3-bromocamphor was used as an electrophile. The corresponding C5-alkylated uridine derivative was obtained as the only recovered product, in low yield (23%), besides the unreacted substrate.

**Scheme 2:**
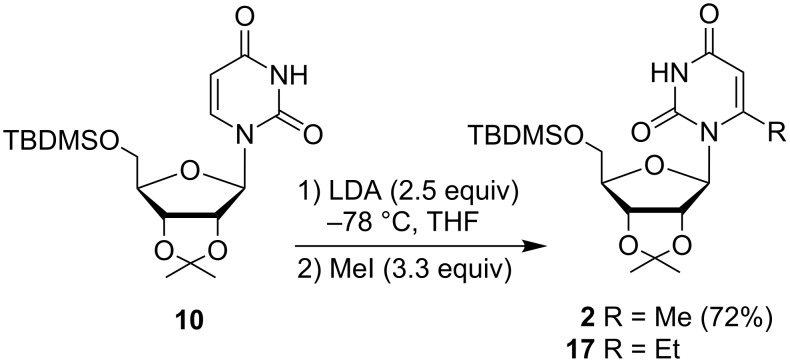
Preparation of 2',3'-*O*-isopropylidene-5'-*O*-(*tert*-butyldimethylsilyl)-6-methyluridine (**2**).

Having these precedents in mind, we decided to investigate the preparation of 6-ω-alkenyluridines **3** by lithiation of 6-methyluridine derivative **2**. Miyasaka et al. observed concomitant formation of 6-ethyl derivative alongside the expected 6-methyl derivative when 2',3'-*O*-isopropylideneuridine (**6**) was allowed to react with LDA and treated with MeI [13]. We found similarly that **10** in the presence of LDA (2.5 equiv) followed by addition of MeI (3.3 equiv) at −78 °C gave a mixture of 6-methyluridine **2** (44%) and 6-ethyluridine **17** (17%) (Scheme 2). By slow addition of the preformed dianion **11** to a THF solution of MeI (reverse-addition mode) [30–31], **2** was produced in satisfactory yield (72%) while formation of 6-ethyluridine **17** was reduced to <5%. Apparently LDA does not coordinate with the substrate in this transformation [32]. 6-Methyluridine can also be synthesized from 5'-*O*-(*tert*-butyl-dimethylsilyl)-6-iodo-2',3'-*O*-isopropylideneuridine via palladium-catalyzed cross-coupling with Me_4_Sn [33].

**Scheme 3:**
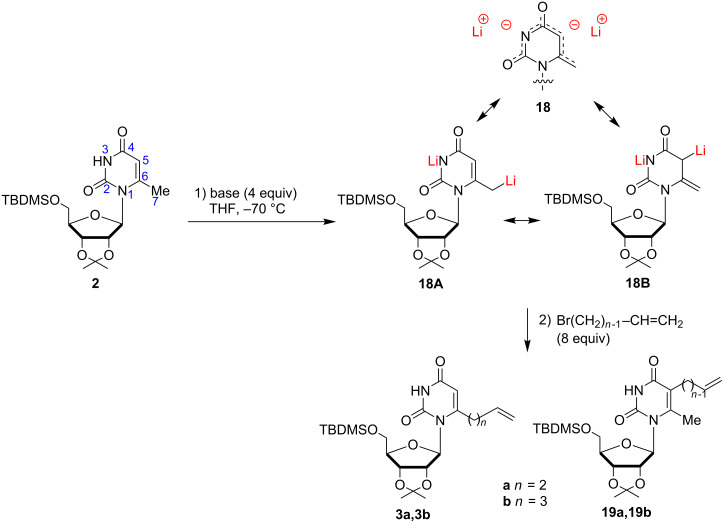
Lateral lithiation/alkylation of 6-methyluridine **2**.

We have then embarked on a detailed investigation of the lithiation/alkylation of 6-methyluridine **2**, varying the base, metalation temperature, and exposure times (Scheme 3). We were concerned with the question of relative acidity of the methyl (C7) and the C5 centers that can compete through **18A** or **18B** [34–36]. In fact the two negative charges at N3 and C5/C7 are delocalized through the O2-C2-N3-C4-O4-C5-C6-C7 bond system rather than being localized dianions: All these forms are resonance structures of the same extended dianionic enolate **18** [37].

In the past, the regioselectivity of reactions of allyl anions have sometimes been explained using the HSAB theory [34]. In the present case, soft electrophiles (ω-alkenyl bromides) are used in the alkylation reaction. However, it is not straightforward to predict the softest center of **18**. In the literature, the regioselectivity of lithiation of allyl anions substituted by one nitrogen at the central carbon (C=C(N)–C) has scarcely been studied [38–40]. Deprotonation of simple enamines or allylamines employing *n*-BuLi and *t*-BuLi/*t*-BuOK produced nitrogen-substituted allylic anions which undergo protonation, alkylation, trimethylsilylation and reaction with carbonyl compounds and epoxides either exclusively or predominantly at the *γ*-position [41–43]. Previous work also showed that cyclic enaminoketones, esters and nitriles were converted into their enolate with *n*-BuLi and alkylated with a variety of alkylating agents, affording the product of an exclusive γ-alkylation [42–45].

The results are summarized in Table 1. An excess of LDA (4 equiv) at −70 °C produced a dilithium reagent, which was presumed to be **18**, as a yellow solution. The colour faded when allyl bromide (8 equiv/ −70 °C → rt/12 h) was introduced, providing a mixture of regioisomers **3a** and **19a** which were separated by chromatography (entry 1, 58% and 10% yield, respectively). With LTMP, the delivery of a less acidic conjugated amine (TMP) in the reaction medium could be expected to prevent protonation of lithiated intermediates and thus to allow more efficient trapping by an electrophile [46–47]. Indeed, LTMP gave a better yield, but a slight decrease in the regioselectivity was observed (**3a**/**19a** 65:20). The ^1^H NMR spectrum of **19a** displayed a singlet at 2.31 ppm, attributed to the C7-methyl group, and the disappearance of the H5 signal at 5.57 ppm. 4-Bromobut-1-ene underwent exclusive C7-carbanion alkylation to **3b** in good yield, with no indication of products resulting from C5-alkylation (entries 3 and 4).

**Table 1:** Lateral lithiation/alkylation of 6-methyluridine **2**.

entry	base	*n*	**3a**,**b** (%)	**19a**,**b** (%)	others (%)

1	LDA	2	58	10	—
2	LTMP	2	65	20	—
3	LDA	3	44	0	—
4	LTMP	3	56	0	—
5	LiHMDS	2	0	0	**20** (40), **21** (18)
6	*s*-BuLi/TMEDA	3	38	0	—

**Figure 4:**
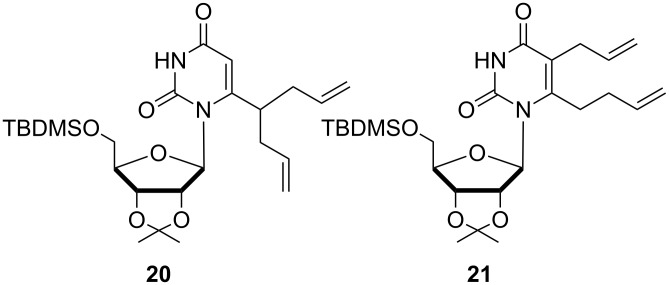
Bis-allylated products **20** and **21**.

Allyl bromide is a good electrophile that can react with both mesomeric forms **18A** and **18B** to give **3a** and **19a**. Apparently dianion **18B** is not nucleophilic enough to react with 4-bromobut-1-ene to give **19b**, and **3b** is formed exclusively. The lateral alkylation of uridine enolate **18** was best accomplished through use of LDA or LTMP as the carbanion generating species, rather than LiHMDS or *s*-BuLi/TMEDA. The bis-allylated products **20** and **21** (Figure 4) were obtained in 40% and 18% yield, respectively with LiHMDS at −70 °C and quenched with allyl bromide (entry 5). This result suggests the remetalation of **3a** is faster than the destruction of LiHMDS by the excess of allyl bromide. Structure of **20** was confirmed by ^1^H NMR and a two-dimensional COSY experiment, which allowed the assignment of the proton–proton correlations of H7 and the allylic methylene groups. Metalation with *s*-BuLi/TMEDA complex was less efficient although the reaction did not lead to degradation products (entry 6). Ring/internal lithiations of uridine derivatives with *s*-BuLi/TMEDA are usually performed with fully TBDMS-protected ribofuranose nucleosides to allow better regiochemical control and to prevent nucleophilic attack of the base on the sugar moiety [48].

## Conclusion

In summary, a straightforward approach to 6-ω-alkenyluridines **3** from readily available protected uridine **1** is proposed. Whereas direct ring alkylation of 6-lithiated uridine **11** with ω-alkenyl bromides failed, our approach relies on lateral lithiation/alkylation of 6-methyluridine **2**. The total synthesis and biological properties of cyclonucleosides **5** will be reported separately.

## Supporting Information

File 1 Experimental section (preparation and spectral data of compounds).
