# Supplementary material for: Selectivity in C-alkylation of dianions of protected 6-methyluridine
Source: Beilstein J Org Chem. 2011 Sep 6;7:1228–33. doi: 10.3762/bjoc.7.143 (PMC3182432; doi:10.3762/bjoc.7.143)
Supplement: File 1 — Experimental section (preparation and spectral data of compounds). [file Beilstein_J_Org_Chem-07-1228-s001.pdf]

Supporting Information

for

Selectivity in *C*-alkylation of dianions of protected 6-methyluridine

Ngoc Hoa Nguyen[1], Christophe Len[2], Anne-Sophie Castanet[*1], and Jacques Mortier[*1]

Address: Université du Maine and CNRS, Unité de chimie organique moléculaire et macromoléculaire (UMR 6011), Faculté des sciences, avenue Olivier Messiaen, 72085 Le Mans Cedex 9, France, Fax: +33 (0) 243 83 39 02 and Université de technologie de Compiègne, Transformations intégrées de la matière renouvelable, EA 4297 UTC/ESCOM, 1 allée du réseau Jean-Marie Buckmaster, 60200 Compiègne, France

Email: Ngoc Hoa Nguyen - bexiuhn@yahoo.com; Christophe Len - christophe.len@utc.fr; Anne-Sopie Castanet[*] - anne-sophie.castanet@univ-lemans.fr; Jacques Mortier[*] - jacques.mortier@univ-lemans.fr

* Corresponding author

Experimental section (preparation and spectral data of compounds).

NMR spectra were recorded on either a 200 or 400 MHz spectrometer. ^{13}C NMR spectra were obtained with broadband proton decoupling. For spectra recorded in $CDCl_3$, chemical shifts were recorded relative to the internal TMS (tetramethylsilane) reference signal. For DMSO-d_6, chemical shifts are given relative to the solvent signal. IR spectra were recorded on a FT-IR spectrophotometer.

Commercial reagents were used without further purification. Flash column chromatography was performed over silica gel (100-200 mesh). Thin layer chromatography was performed using silica gel F_{254} TLC plates and visualized with UV. Tetrahydrofuran was dried from sodium benzophenone ketyl. Alkyllithium reagents were titrated periodically against *N*-benzylbenzamide [1]. *N,N,N',N'*-Tetramethyl-1,2-ethylenediamine (TMEDA) and 2,2,6,6-tetramethylpiperidine were distilled from CaH_2. Diisopropylamine was distilled from KOH.

2',3'-*O*-isopropylideneuridine (6). To a suspension of uridine (10.00 g, 41.0 mmol) and *p*-toluenesulfonic acid monohydrate (1.07 g, 5.6 mmol) in acetone (50 mL) was added 2,2-dimethoxypropane (25 mL, 201 mmol) at room temperature. The resulting mixture was stirred overnight, the white precipitate was filtered off, washed with acetone, and dried under vacuum to give 2',3'-*O*-isopropylideneuridine (**6**) (9.27 g, 80%) as a white solid (mp 160−161 °C, lit. [2] 161 °C). ^{1}H NMR (200 MHz, $CDCl_3$) δ: 7.83 (d, 1H, J = 8.1 Hz, H6), 5.87 (d, 1H, J = 2.8 Hz, H1'), 5.68 (d, 1H, J = 8.1 Hz, H5), 4.91 (dd, 1H, J = 6.3, 2.8 Hz, H2'), 4.82 (dd, 1H, J = 6.3, 3.4 Hz, H3'), 4.20 (m, 1H, H4'), 3,77 (dd, 1H, J = 11.9, 3.6 Hz, H5'), 3.71 (dd, 1H, J = 4.6, 1.9 Hz, H5"), 1.54 (s, 3H, $C(CH_3)$), 1.35 (s, 3H, $C(CH_3)$). ^{13}C NMR (50 MHz, DMSO-d_6) δ: 164.2, 151.3, 142.9, 114.0, 102.3, 92.2, 87.5, 84.7, 81.5, 62.3, 28.0, 26.2. IR (υ, cm^{-1}): 3240, 2986, 1661, 1465, 1117, 801.

2',3'-*O*-isopropylidene-5'-*O*-TBDMS-uridine (10). 2',3'-*O*-isopropylideneuridine (**6**) (7.00 g, 24.6 mmol) was dissolved in DMF (100 mL) at rt. Imidazole (3.32 g, 49 mmol), DMAP (1.45 g, 12 mmol) and *tert*-butyldimethylsilyl chloride (8.40 g, 55 mmol) were successively added. After being stirred at rt for 16 h, the reaction mixture was diluted with water (170 mL) and extracted with ethyl acetate (3 × 100 mL). The combined organic layers were washed with brine, dried over $MgSO_4$, filtered, and concentrated *in vacuo*. Chromatography on silica gel (CH_2Cl_2/MeOH 95:5) afforded 2',3'-*O*-isopropylidene-5'-*O*-TBDMS-uridine (**10**) (9.29 g, 95%) as a white solid (mp 135−136 °C, lit. [3] 134−135 °C). ^{1}H NMR (200 MHz, $CDCl_3$) δ: 7.70 (d, 1H, J = 8.1 Hz, H6), 5.99 (d, 1H, J = 2.8 Hz, H1'), 5.69 (d, 1H, J = 8.1 Hz, H5), 4.77 (dd, 1H, J = 6.2, 2.9 Hz, H2'), 4.68 (dd, 1H, J = 6.2, 2.7 Hz, H3'), 4.33 (m, 1H, H4'), 3,94 (dd, 1H, J = 11.5, 2.4 Hz, H5'), 3.80 (dd, 1H, J = 11.5, 3.0 Hz, H5"), 1.60

(s, 3H, $C(CH_3)$), 1.36 (s, 3H, $C(CH_3)$), 0.91 (s, 9H, $C(CH_3)_3$), 0.10 (s, 6H, CH_3). ^{13}C NMR (100 MHz, $CDCl_3$) δ: 163.7, 150.3, 140.7, 114.1, 102.2, 91.9, 86.7, 85.3, 80.3, 63.4, 27.3, 26.0, 25.7, 18.5, -5.5. IR (υ, cm^{-1}): 2935, 2855, 1684, 1461, 1268, 1125, 829.

2',3'-*O*-isopropylidene-5'-*O*-TBDMS-6-deuterouridine (12). 2',3'-*O*-isopropylidene-5'-*O*-TBDMS-uridine (**10**) (0.825 g, 2.07 mmol) in THF (10 mL) was added dropwise to a solution of LDA (10 mmol) in THF (10 mL) under an argon atmosphere at −70 °C. The resulting mixture was stirred for 1 h, treated with D_2O (0.2 mL, 14.5 mmol) and stirred for an additional hour at −70 °C. The solution was warmed gradually to rt, hydrolyzed with water (10 mL), the aqueous layer was extracted with ethyl acetate (3 × 15 mL), and the combined organic layers were washed with brine, dried over $MgSO_4$, filtered and concentrated *in vacuo*. The residue was purified by column chromatography on silica gel (cyclohexane/ethyl acetate 8:2 → 6:4) to give 2',3'-*O*-isopropylidene-5'-*O*-TBDMS-6-deuterouridine (**12**) in 82% yield. ^{1}H NMR (400 MHz, $CDCl_3$) δ: 5.99 (d, 1H, J = 2.8 Hz, H1'), 5.69 (s, 1H, H5), 4.77 (dd, 1H, J = 6.2, 2.9 Hz, H2'), 4.68 (dd, 1H, J = 6.2, 2.7 Hz, H3'), 4.33 (m, 1H, H4'), 3,94 (dd, 1H, J = 11.5, 2.4 Hz, H5'), 3.80 (dd, 1H, J = 11.5, 2.9 Hz, H5"), 1.60 (s, 3H, $C(CH_3)$), 1.36 (s, 3H, $C(CH_3)$), 0.91 (s, 9H, $C(CH_3)_3$), 0.10 (s, 6H, CH_3).

2',3'-*O*-isopropylidene-5'-*O*-TBDMS-6-methyluridine (2). 2',3'-*O*-isopropylidene-5'-*O*-TBDMS-uridine (**10**) (1.184 g, 2.97 mmol) in THF (12 mL) was added dropwise to a solution of LDA (7.5 mmol) in THF (15 mL) under argon at −78 °C. After 3 h at −78 °C, the resulting solution was added to a solution of iodomethane (0.6 mL, 9.6 mmol) in THF (12 mL) at −78 °C. Stirring was maintained for 1 h and the solution was allowed to warm to rt. Water (20 mL) was added, the aqueous phase was extracted with ethyl acetate, and the combined organic layers were dried over $MgSO_4$, filtered and concentrated *in vacuo*. The residue was purified by column chromatography on silica gel (cyclohexane/ethyl acetate 8:2 → 6:4) to give 2',3'-*O*-isopropylidene-5'-*O*-TBDMS-6-methyluridine (**2**) (890 mg, 72%) as a white solid. (mp 120 °C). ^{1}H NMR (400 MHz, $CDCl_3$) δ: 9.26 (broad s, 1H, NH), 5.71 (d, 1H, J = 1.3 Hz, H1'), 5.57 (s, 1H, H5) 5.22 (dd, 1H, J = 6.3, 1.3 Hz, H2'), 4.82 (dd, 1H, J = 6.3, 4.5 Hz, H3'), 4.14 (m, 1H, H4'), 3.81 (m, 2H, H5'and H5"), 2,34 (s, 3H, CH_3), 1.54 (s, 3H, $C(CH_3)$), 1.34 (s, 3H, $C(CH_3)$), 0.88 (s, 9H, $C(CH_3)_3$), 0.04 (s, 6H, CH_3). ^{13}C NMR (100 MHz, $CDCl_3$) δ: 163.5, 153.3, 150.8, 113.7,

103.0, 91.7, 89.7, 84.3, 82.0, 64.3, 27.3, 25.3, 20.4, 18.5, -5.2. IR (υ, cm^{-1}): 2929, 2858, 1701, 1387, 1092, 837. HRMS calcd for $C_{15}H_{23}N_2O_6Si$ ([M-C_4H_9]): 355.1325, found 355.1310.

Metalation of 2',3'-*O*-isopropylidene-5'-*O*-TBDMS-6-methyluridine (2) – General procedure. To a solution of the base (3 mmol) in THF (4 mL) at −70 °C was added dropwise 2',3'-*O*-isopropylidene-5'-*O*-TBDMS-6-methyluridine (**2**) (0.75 mmol) in THF (4 mL). Stirring was maintained 1 h at −70 °C. The solution was quenched with the electrophile (8 equiv), stirred at −70 °C for 30 min and then gradually warmed to ambient temperature. Water (5 mL) was added, the aqueous phase was extracted with ethyl acetate. The combined organic layers were dried over $MgSO_4$, filtered, and concentrated *in vacuo*. The residue was purified by column chromatography on silica gel (cyclohexane/ethyl acetate 9:1 → 6:4).

2',3'-*O*-isopropylidene-5'-*O*-TBDMS-6-but-3-enyluridine (3a). Pale yellow viscous oil (65%, entry 2, Table 1). ^{1}H NMR (400 MHz, $CDCl_3$) δ: 9.35 (broad s, 1H, NH), 5.81 (ddt, 1H, J = 16.8, 10.2, 6.6 Hz, C*H*=CH_2), 5.69 (s, 1H, H1'), 5.57 (d, 1H, J = 2.3 Hz, H5), 5.21 (dd, 1H, J = 6.6, 1.3 Hz, H2'), 5.18-5.10 (m, 2H, CH=CH_2), 4.81 (dd, 1H, J = 6.3, 4.5 Hz, H3'), 4.15 (m, 1H, H4'), 3.86-3.78 (m, 2H, H5' and H5"), 2,68 (t, 2H, J = 7.1 Hz, CH_2), 2.41 (m, 2H, CH_2), 1.54 (s, 3H, C(CH_3)), 1.34 (s, 3H, C(CH_3)), 0.88 (s, 9H, C$(CH_3)_3$), 0.05 (s, 6H, CH_3). ^{13}C NMR (50 MHz, $CDCl_3$) δ: 163.5, 156.0, 150.8, 135.1, 117.0, 113.7, 102.2, 91.5, 89.6, 84.3, 82.1, 64.3, 32.3, 31.3, 27.3, 25.9, 25.3, 18.4, -5.2. HRMS calcd for $C_{18}H_{27}N_2O_6Si$ ([M-C_4H_9]): 395.1638, found 395.1593.

2',3'-*O*-isopropylidene-5'-*O*-TBDMS-5-allyl-6-methyluridine (19a). Pale yellow viscous oil (20%, entry 2, Table 1). ^{1}H NMR (400 MHz, $CDCl_3$) δ: 9.34 (broad s, 1H, NH), 5.84-5.72 (m, 2H, H1' and C*H*=CH_2), 5.22 (d, 1H, J = 6.3 Hz, H2'), 5.02 (dd, 1H, J = 10.1, 1.5 Hz, CH=CH_2), 5.00 (dd, 1H, J = 16.9, 1.5 Hz, CH=CH_2), 4.83 (dd, 1H, J = 6.3, 4.5 Hz, H3'), 4.13 (m, 1H, H4'), 3.85-3.78 (m, 2H, H5' and H5"), 3.20 (m, 2H, CH_2) 2,31 (s, 3H, CH_3), 1.53 (s, 3H, C(CH_3)), 1.33 (s, 3H, C(CH_3)), 0.87 (s, 9H, C$(CH_3)_3$), 0.03 (s, 6H, CH_3). ^{13}C NMR (100 MHz, $CDCl_3$) δ: 163.2, 150.2, 149.6, 134.4, 115.3, 113.5, 110.9, 92.0, 89.5, 84.3, 81.9, 64.2, 29.3, 27.1, 25.9, 25.3, 18.4, 16.4, -5.3. IR (υ, cm^{-1}): 2928, 2856, 1680, 1461, 1252, 1062. HRMS calcd for $C_{18}H_{27}N_2O_6Si$ ([M-C_4H_9]): 395.1638, found 395.1652.

2',3'-*O*-isopropylidene-5'-*O*-TBDMS-6-pent-4-enyluridine (3b). Pale yellow viscous oil (56%, entry 4). ^{1}H NMR (400 MHz, $CDCl_3$) δ: 9.28 (broad s, 1H, NH), 5.79 (ddt, 1H, J = 17.2, 10.4, 6.5 Hz, C*H*=CH_2), 5.66 (s, 1H, J = 1.0 Hz, H1'), 5.56 (d, 1H, J = 2.0 Hz, H5), 5.19 (dd, 1H, J = 6.4, 1.0 Hz, H2'), 5.12-5.04 (m, 2H, CH=CH_2), 4.80 (dd, 1H, J = 6.5, 4.5 Hz, H3'), 4.15 (m, 1H, H4'), 3.85-3.77 (m, 2H, H5' and H5"), 2,57 (t, J = 7.8 Hz, 2H, CH_2), 2.18 (m, 2H, CH_2), 1.75 (m, 2H, CH_2), 1.54 (s, 3H, C(CH_3)), 1.33 (s, 3H, C(CH_3)), 0.87 (s, 9H, C$(CH_3)_3$), 0.04 (s, 6H, CH_3). ^{13}C NMR (50 MHz, $CDCl_3$) δ: 163.5, 156.7, 150.8, 136.7, 116.3, 113.6, 102.0, 91.4, 89.6, 84.3, 82.1, 64.2, 32.6, 30.0, 27.2, 26.6, 25.8, 25.3, 18.4, -5.3. IR (υ, cm^{-1}): 2929, 2857, 1687, 1381, 1082, 1060. HRMS calcd for $C_{19}H_{29}N_2O_6Si$ ([M-C_4H_9]): 409.1795, found 409.1793.

2',3'-*O*-isopropylidene-5'-*O*-TBDMS-6-(1-allyl-but-3-enyl)uridine (20). Pale yellow viscous oil (40%, entry 5). ^{1}H NMR (400 MHz, $CDCl_3$) δ: 8.80 (broad s, 1H, NH), 5.79 (s, 1H, H'-1), 5.69 (m, 2H, C*H*=CH_2), 5.55 (d, 1H, J = 2.3 Hz, H5), 5.18 (dd, 1H, J = 6.4, 1.1 Hz, H2'), 5.19-5.08 (m, 4H, CH=CH_2), 4.80 (dd, 1H, J = 6.4, 4.5 Hz, H3'), 4.15 (m, 1H, H4'), 3.84-3.77 (m, 2H, H5' and H5"), 2.95 (quint., 1H, J = 6.3 Hz, CH), 2.46-2.33 (m, 4H, CH_2), 1.54 (s, 3H, C(CH_3)), 1.33 (s, 3H, C(CH_3)), 0.87 (s, 9H, C$(CH_3)_3$), 0.04 (s, 6H, CH_3). ^{13}C NMR (100 MHz, $CDCl_3$) δ: 163.5, 158.8, 150.9, 133.7, 133.2, 118.8, 118.4, 113.6, 101.1, 90.9, 89.7, 84.5, 82.0, 64.3, 37.4, 36.4, 27.2, 25.8, 25.3, 18.4, -5.3. IR (υ, cm^{-1}): 2929, 2857, 1682, 1383, 1081, 1060. HRMS calcd for $C_{25}H_{41}N_2O_6Si$ ([M+H$^+$]): 493.2734, found 493.2712.

2',3'-*O*-isopropylidene-5'-*O*-TBDMS-5-allyl-6-(but-3-enyl)uridine (21). Pale yellow viscous oil (18%, entry 5). ^{1}H NMR (400 MHz, $CDCl_3$) δ: 8.94 (broad s, 1H, NH), 5.86-5.71 (m, 2H, C*H*=CH_2), 5.64 (s, 1H, H1'), 5.19-5.12 (m, 2H, =CH_2), 5.09-4.96 (m, 3H, H2' and =CH_2), 4.77 (dd, 1H, J = 6.4, 4.5 Hz, H3'), 4.15 (m, 1H, H4'), 3.85-3.76 (m, 2H, H5' and H5"), 3.18 (m, 2H, CH_2), 2.71 (m, 2H, CH_2), 2.37 (m, 2H, CH_2), 1.54 (s, 3H, C(CH_3)), 1.34 (s, 3H, C(CH_3)), 0.87 (s, 9H, C$(CH_3)_3$), 0.04 (s, 6H, CH_3). ^{13}C NMR (100 MHz, $CDCl_3$) δ: 163.1, 152.5, 150.2, 135.4, 134.9, 116.7, 115.6, 113.8, 110.7, 92.4, 89.5, 84.4, 82.2, 64.3, 31.9, 29.6, 28.5, 26.0, 25.4, 18.5, -5.2. IR (υ, cm^{-1}): 2929, 2856, 1681, 1381, 1083, 1061 HRMS calcd for $C_{25}H_{41}N_2O_6Si$ ([M+H$^+$]): 493.2734, found 493.2722.

References and notes

1. Burchat, A. F; Chong, J. M.; Nielsen, N *J. Organomet. Chem.* **1997**, *542*, 281-283.
2. Chabaud, P.; Camplo, M.; Payet, D.; Serin, G.; Moreau, L.; Barthelemy, P.; Grinstaff, M. W. *Bioconjugate Chem.* **2006**, *17*, 466-472.
3. Zhang, X.; Bernet, B.; Vasella, A. *Helv. Chim. Acta* **2006**, *89*, 2861-2917.
